# One-Step Agrobacterium Mediated Transformation of Eight Genes Essential for Rhizobium Symbiotic Signaling Using the Novel Binary Vector System pHUGE

**DOI:** 10.1371/journal.pone.0047885

**Published:** 2012-10-24

**Authors:** Andreas Untergasser, Gerben J. M. Bijl, Wei Liu, Ton Bisseling, Jan G. Schaart, René Geurts

**Affiliations:** 1 Laboratory of Molecular Biology, Department of Plant Science, Wageningen University, Wageningen, The Netherlands; 2 Wageningen UR Plant Breeding, Wageningen University and Research Centre, Wageningen, The Netherlands; University of Nottingham, United Kingdom

## Abstract

Advancement in plant research is becoming impaired by the fact that the transfer of multiple genes is difficult to achieve. Here we present a new binary vector for *Agrobacterium tumefaciens* mediated transformation, pHUGE-Red, in concert with a cloning strategy suited for the transfer of up to nine genes at once. This vector enables modular cloning of large DNA fragments by employing Gateway technology and contains DsRED1 as visual selection marker. Furthermore, an R/Rs inducible recombination system was included allowing subsequent removal of the selection markers in the newly generated transgenic plants. We show the successful use of pHUGE-Red by transferring eight genes essential for *Medicago truncatula* to establish a symbiosis with rhizobia bacteria as one 74 kb T-DNA into four non-leguminous species; strawberry, poplar, tomato and tobacco. We provide evidence that all transgenes are expressed in the root tissue of the non-legumes. Visual control during the transformation process and subsequent marker gene removal makes the pHUGE-Red vector an excellent tool for the efficient transfer of multiple genes.

## Introduction

Genetically modified plants are irreplaceable tools in plant science and have the potential to revolutionize agriculture. Genetic modification allows improving their nutritional value, adapting them to hostile environments, enabling production of raw industrial chemicals, providing resistance to diseases, enhancing fertilizer uptake and preparing them for the production of medicinal products including vaccines and pharmaceuticals. A long-standing goal is the transfer of the ability to establish a symbiosis with nitrogen-fixing rhizobia to non-legume crop species, making them independent of nitrogen fertilization [1]–[2]. Most of such genetic modifications require transfer of more than one gene, up to the transfer of complete pathways.

Transgenic plants can be produced by viral transformation [3], electroporation of protoplasts [4], particle bombardment [5] and *Agrobacterium*-mediated transformation [6], with the latter being the most popular method. *Agrobacterium*-mediated transformation was developed shortly after it had been elucidated that crown gall tumor genesis was caused by the introduction of a bacterial DNA fragment into the plant genome [6] (see [7] for a review on *Agrobacterium* transformation). Today, a broad range of transformation systems for *Agrobacterium* is available, with some binary vectors optimized for special needs. For example some vectors, e. g. BIBAC and pYLTAC7, were designed for high stability of big constructs by employing low copy amplification in *Escherichia coli* (*E. coli)* as well as in *Agrobacterium* [8]–[9]. Such vectors allow the transfer of large DNA fragments up to 150 kb into plant genomes. Other vectors allow the removal of marker genes from the plant genome after transformation by using a yeast or phage recombinase system [10]–[12]. In the pMF1 vector for example the marker genes and a synthetic R recombinase gene are flanked by Rs recombination sites. The synthetic R recombinase can be activated by dexametasone, by which it translocates from the cytoplasm into the nucleus of the plant cells. Subsequently, it recombines the Rs sites thereby removing the marker genes from the genome [10]–[11]. Finally, many vectors today are available with introduced gateway cassettes allowing easy cloning and thereby removing the need to work with rare cutting enzymes as well as enabling MultiRound Gateway technology to clone several genes in one binary vector [13]–[15].

Legumes have a rather unique character to establish a root nodular symbiosis with nitrogen-fixing *Rhizobium*. With the development of legume model species like Medicago (*Medicago truncatula*) and Lotus (*Lotus japonicus*) insight in the genetic networks that control this symbiosis has increased markedly. Central in the legume-*Rhizobium* symbiosis is plant perception of the bacterial lipochitooligosaccharide signal molecule, known as nodulation (Nod) factor. Perception of this signal and the subsequent induced signaling is essential for nodule formation and bacterial infection (for review see: [16]–[17]). Genetic studies in both model legumes revealed that the Nod factor signaling network is made up by seven to eight key genes that are constitutively expressed in roots and are essential to trigger almost all symbiotic responses, including Nod factor induced gene expression. Mutants affected in these genes do not display any obvious non-symbiotic phenotype, suggesting that in legumes these genes are primarily involved in symbiotic signaling. It is very probable that these Nod factor signaling proteins alone are insufficient to form a functional biochemical signaling network. More likely they will form such a network together with more generic proteins encoded by additional genes. These remaining genes are likely not symbiosis specific and we speculate that these genes are also functional in non-legumes. Therefore we hypothesize, that the introduction of a key set of legume Nod factor signaling genes in a non-legume could be sufficient to establish a functional signaling network.

A project of these dimensions demands a transfer vector with special skills, as for this project eight genes need to be transferred. First, the vector should allow the transfer of many genes in a single transformation step. Although several genes can be transferred by cotransformation, this approach might come to its limitations if eight genes need to be integrated. The alternative of crossing plants with single gene transformations is also highly impractical, due to the long generation time of poplar, for example, and the large number of crossings required by the eight genes transferred. Second, due to the size of the eight genes including promoters and terminators, the vector needs to stabilize large T-DNAs in *E. coli* as well as *Agrobacterium* and be capable of their transfer. Third, due to the possibility of essential genes missed out in the first transformation experiments, the vector should allow marker removal thereby enabling re-transformation of obtained plants. Fourth, the transformed plants should be easily traceable by an expressed visual (fluorescent) marker. Finally, the cloning into the vector should enable Gateway technology and circumvent restriction based cloning, which is impaired by the availability of unique restriction sites and low efficiency using large plasmids. Today, none of the available binary vectors fulfills all these requirements, therefore we constructed two new binary plasmids, pHUGE-Red and pHUGE-RedSeed.

Here we present the successful use of this vector system by transforming four non-leguminous species, strawberry, poplar, tomato and tobacco, with eight legume Nod factor signaling genes. We aim to test whether it is possible to activate marker gene expression in non-legumes upon Nod factor application.

## Materials and Methods

### Construction of pHUGE-Red Plasmids

Most cloning steps were performed in *E. coli* strain DH5α. Plasmids larger than 35 kb were transformed using commercial library competent E. coli strain DH10B (Invitrogen, Carlsbad, California). DEST plasmids were grown in *E. coli* strain DB3.1. Transformations resulted in average in 80–500 colonies per ligation or per Gateway reaction. Of each plate 20 colonies were selected for further analysis with 80–100% carrying the desired construct. Results of the *att* site removal experiment were comparable. The construction of pHUGE-Red (accession nr.: JN874480) and pHUGE-RedSeed (accession nr.: JN874481) is described in detail in Data S1. Plasmids are freely available for not-for-profit entities for research purposes at the Functional Genomics unit of the Department of Plant Systems Biology (https://gateway.psb.ugent.be/search, search for pHUGE). Construction of pHUGE-MtNFS (accession nr.: JN874482) and pHUGE-LjMtNFS (accession nr.: JN874483) is described in the results section.

### Transformation of Plants and Removal of Marker Genes

*Agrobacterium tumefaciens* strain AGL1 (recA-) was transformed using electroporation and successful transformation was confirmed by Southern blot analysis. Plants were transformed as published: strawberry (*Fragaria×ananassa* var. Calypso) [18], tobacco (*Nicotiana tabacum* var. Samsun) [19], tomato (*Solanum esculentum* var. Moneymaker) see www.hos.ufl.edu/meteng/HansonWebpagecontents/Tomatotransformatioprotocol.html, poplar (*Populus tremula × P. alba hybrid* clone 7171B4) [20] and Arabidopsis (*Arabidopsis thaliana* var. Columbia) [21]. Marker genes embedded between Rs recombination sites were removed as described by Schaart et al. [11].

### Verification of Marker Removal by PCR Analysis

Two primer pairs were designed, using Primer3plus [22], to confirm successful marker removal by recombination (Table S1). Pair 1 consists of REC-UNI-f, positioned in the Left Border region, in combination with REC-IN-r, positioned inside the recombination cassette. This pair can be used to test for the presence of the marker genes cassette. An amplicon of 553 bp will be obtained in case the marker cassette is present. A second reverse primer, REC-OUT-r, is positioned downstream of the marker cassette, and can be used in combination with REC-UNI-f to verify successful recombination. In case recombination has occurred, the amplicon fragment size is 503 bp, whereas when the cassette is still present the amplicon will be 10,150 bp.

### RNA Isolation Method

50 mg of deep-frozen plant material is homogenized in liquid nitrogen, 500 µl Trizol is added and subsequently after 5 min incubation chloroform extracted. The RNA is precipitated by adding 70% v/v ethanol. This mixture is transferred onto an RNeasy mini DNA column (Qiagen, Hilden, Germany). Remainder of procedure is performed according to manufacturer recommendations including on column DNAse treatment.

### cDNA Synthesis

cDNA was synthesized from 1 µg of total RNA using SuperScript II according to manufacturer protocol (oligo(dT) priming) (Invitrogen, Carlsbad, California).

### Quantitative RT-PCR

Quantitative RT-PCR has been performed using chemicals supplied by Eurogentec (Maastricht, the Netherlands). Experimental setup and execution have been conducted using a MyIQ (Biorad, Hercules, USA) optical cycler, according to protocol provided by manufacturer. Sequences of primers used can be found in Table S1.

### Fluorescence Microscopy

Imaging of DsRED1 was done using the Leica MZIII fluorescence stereomicroscope with the appropriate filter settings (excitation 565/30, emission 620/60; Filter by AHF Analysetechnik AG, Tübingen, Germany; F91-701, F44-021, F42-010). Images were processed electronically.

### Analysis of Promoter GUS Reporter Constructs

The upstream promoter regions of the Medicago Nod factor signaling genes MtNFP, MtLYK3, MtDMI1, MtDMI2, MtDMI3, MtNSP1 and MtNSP2 have been cloned into pENTR-D/TOPO (Invitrogen, Carlsbad, California) according to manufacturer protocol. Destination clones were made using Gateway technology by recombining each of the promoters into binary vector pKGWFS7-RR containing a GUS-GFP fusion reporter as well as pAtUBQ10::DsRed1 as a selectable marker [15] [23]–[24]. These vectors have been introduced into three different plants species; Medicago, Arabidopsis and tomato using *A. rhizogenes* based transformation as described by Limpens et al [23]. Compound transformed plants were grown on Farhaeus medium [25] with reduced nitrate (2,5 mM) for three days. Afterwards roots were harvested for staining. GUS staining was performed according to Jefferson et al. [26] with few modifications. Plant material was incubated in 0.05% (w/v) 5-bromo-4-chloro-3-indolyl-b-glucuronic acid (Duchefa, Haarlem, The Netherlands) in 0.1 M sodium phosphate buffer (pH 7) with 3% Sucrose, 5 mM potassium-ferrocyadine and 5 mM potassium-ferricyanide. Vacuum was applied for 1 h, and the samples were incubated at 37°C. For imaging, a Nikon (Tokyo, Japan) SMZ-U stereomicroscope and a Nikon optiphot-2 bright field microscope were used. Photographs were made using a Nikon coolpix990 digital camera.

### Nodulation Assay

For the complementation assay of Medicago mutants with the respective genes *A. rhizogenes* transformation was used [23]. Compound plants with transgenic roots were transferred to perlite soaked in Fahraeus medium [25] containing *Sinorhizobium meliloti* (*S. meliloti*) strain 2011 (OD600 = 0.05). Plants were scored for nodulation 21 days post inoculation.

For the nodulation assay of transgenic non-legumes plants with were transferred to perlite soaked in Fahraeus medium [25] containing *S. meliloti* strain 2011 or *Sinorhizobium* sp. NGR234 induced with 1.5 µM Luteolin (*S. meliloti* strain 2011) or Naringenin (*Sinorhizobium* sp. NGR234), applied at OD600 = 0.05. Plants were scored for nodulation 21 days post inoculation.

### Root Hair Deformation Assay

Seeds were germinated on 9 cm 1% water Daishin agar plates and grown to 1 cm size. Root hair deformation assays were performed on square 12 cm plates as well as Fahraeus slides [27]. In both cases plants were grown in BNM (in case of plate assay solidified with 1% Daishin agar) [28]. Root hair deformation was investigated 3, 6 and 12 hour post application of the appropriate Nod factor mixtures (applied at ∼10^−9^ M) that were extracted from *Sinorhizobium* sp. NGR234 and *S. meliloti* [29]–[30].

## Results

### Construction of Binary pHUGE-Red Vectors

**Figure 1:**
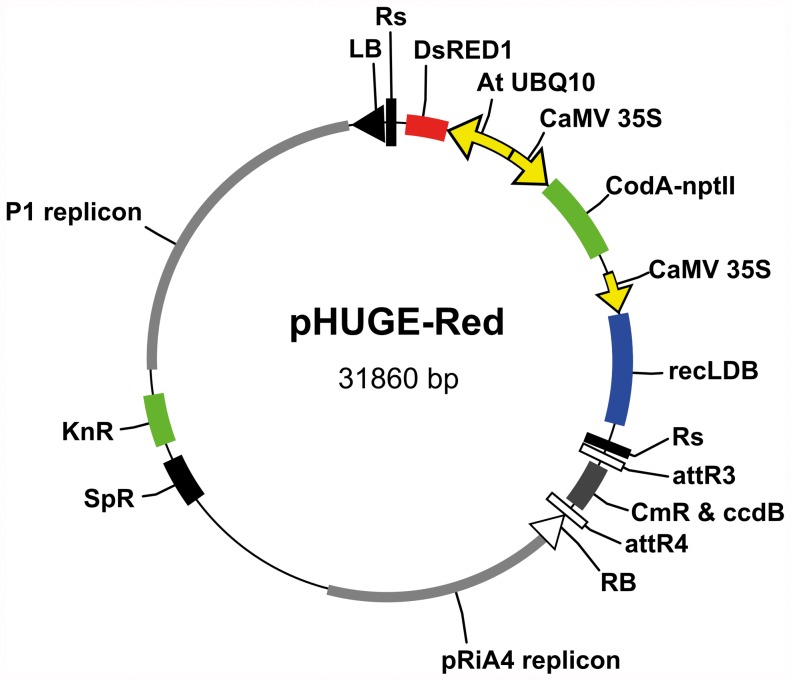
Physical map of binary vector pHUGE-Red. Indicated are left border (LB), right border (RB), Rs-recombination sites (Rs), R-recombinase-LDB fusion gene (recLDB), the visual marker *DsRED1* (DsRED1) driven by *AtUBQ10* promoter, the fused selection markers *CodA-nptII* under control of *CaMV35S* promoter and the components required for multisite gateway; recombination sites (*attR3* and *attR4*) and two selection markers (CmR and ccdB). The backbone contains the P1 replicon as well as the pRiA4 replicon. Selection markers: kanamycin (KnR) and spectinomycin (SpR). In vector pHUGE-RedSeed the *AtUBQ10* promoter was exchanged for the seed coat specific napin promoter. Complete sequences are available at the GenBank database (pHuge-Red accession no. JN874480, pHuge-RedSeed accession no. JN874481).

Vector pYLTAC7, previously successfully used in transfer of BAC-sized T-DNA inserts [9] was taken as a starting point. The vector was made compatible for MultiSite Gateway by introducing the spectinomycin resistance gene (*SpR*) in the backbone of the plasmid and cloning of an *attR4*-*attR3* cassette, containing a chloramphenicol resistance gene (*CmR*) and a *ccdB* gene, between the left and the right border sequences [31]. For selection of transgenic plant material two markers were included; a hybrid cytosine deaminase/kanamycin resistance gene *CodA-ntpII* [11], and the red fluorescent protein encoding gene *DsRED1* as a visual marker. In case of the latter we made two variants; *DsRED1* driven by the constitutive *AtUBQ10* promoter of Arabidopsis [23] or the seed coat specific *Brassica napa* napin (*napA*) promoter [32]. To allow removal of both selection markers after successful plant transformation, we flanked these markers with Rs recombination sites and included a gene encoding an R recombinase fused to the ligand binding domain (LDB) of the rat glucocorticoid receptor. This gene fusion is driven by the constitutive *CaMV 35S* promoter and the subsequent fusion protein can be activated post-translationally by application of dexamethasone [11]. *CodA-ntpII* acts as a negative selection marker in presence of 5-fluorocytosine thereby facilitating selection of lines in which the marker cassette has been removed [11]. The stepwise method of cloning is provided as Data S1. The two resulting binary vectors were named pHUGE-Red (accession nr.: JN874480) and pHUGE-RedSeed (accession nr.: JN874481), respectively (Fig. 1).

### Selection of Legume Genes

To test the functionality of the pHUGE-Red binary vector system we aimed to transfer legume Nod factor signaling genes to non-legume species. We selected seven Medicago genes that form the core genetic signaling network; *MtNFP*, *MtLYK3*, *MtDMI1*, *MtDM2*, *MtDMI3*, *MtNSP1* and *MtNSP2* [33]–[37]. The LysM receptor kinases named LjNFR5*/*MtNFP and LjNFR1/MtLYK3 in Lotus and Medicago are involved specifically in perception of Nod factors of their particular host rhizobial strain [38]–[39] [33]–[34]. In case of Lotus Nod factors have to be fucosylated at the reducing end of the chitooligosaccharide backbone, whereas in case of Medicago Nod factors are sulphated at this position. Adjustment of host range specificity by the introduction of foreign Nod Factor receptors has been shown previously [39]. As the broad host range strain *Sinorhizobium* sp. NGR234 produces a mixture of Nod factors among some are fucosylated, but not solely sulphated [29], we decided to make also a second binary construct by replacing the Medicago *MtNFP* and *MtLYK3* by Lotus *LjNFR1* and *LjNFR5*. The remaining genes encode proteins that function in or around the nucleus; among these genes are two GRAS-type transcriptional regulators MtNSP1 and MtNSP2 essential for transcriptional activation of Nod factor induced genes [36]–[37].

Besides Nod factor signaling genes that are constitutively expressed prior to Nod factor perception we included *MtNIN* as a reporter gene. In legume roots basal expression of MtNIN is relatively low and, upon rhizobium application the level of MtNIN expression is strongly induced within several hours in a Nod factor signaling network dependent manner [40]–[42]. The (putative) transcription factor MtNIN is, besides a good marker, also an essential component for Nod factor induced nodule organogenesis [40]–[41] [43].

### Cloning and Expression Control of Legume Genes

**Figure 2:**
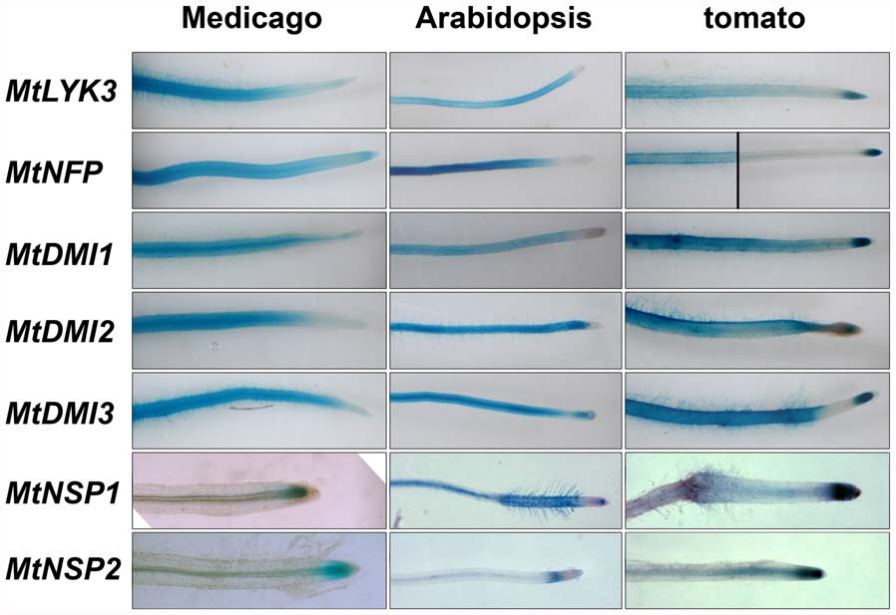
GUS reporter studies in legumes and non-legumes. Histochemical blue staining of roots from Medicago (left row), Arabidopsis (center row) and tomato (right row) transformed with different Medicago Nod factor signaling gene promoter::GUS constructs.

We aimed to clone all eight genes in their genomic context, including approximately 2–3 kb of regulatory regions up- and downstream of the coding sequence. To validate functioning of these regulatory regions the cloned genes were tested for functional complementation of the corresponding Medicago mutants using *Agrobacterium rhizogenes* based root transformation. Upon inoculation with *S. meliloti* nodules were formed on transgenic roots for each of the genes tested. Zero nodules were found on untransformed mutants or mutants transformed with an empty vector. Nodule numbers of the complemented plants are comparable to the number of nodules on control plants (Table 1). This shows that the selected genomic regions contain all essential regulatory elements to drive the corresponding gene adequately in legumes. To determine whether these promoter regions maintain functionality when transferred to non-legumes, GUS-reporter constructs for seven Medicago genes were constructed. We selected two non-legume species that are phylogenetically rather distinct from legumes; namely tomato (*Solanum esculentum*) and Arabidopsis. Both species can be efficiently transformed using *A. rhizogenes*. In Medicago roots, promoter activity of the *MtLYK3*, *MtNFP*, *MtDMI1*, *MtDMI2* and *MtDMI3* regulatory regions was detected in all cell layers of the differentiation- and, young mature zone, whereas the regulatory regions of *MtNSP1* and *MtNSP2* are most active in meristematic region (Fig. 2). Introduction of the GUS-reporter constructs in tomato roots revealed that the transcriptional activity of all seven genes largely overlapped to the spatial pattern found in Medicago (Fig. 2). Additionally however, activity of the *MtNFP*, *MtLYK3*, *MtDMI1*, *MtDMI2* and *MtDMI3* promoter regions was found also in the root meristematic zone of tomato, whereas this is not the case for Medicago. Based on the largely overlapping spatial expression patterns of all seven genes in tomato and Medicago roots we decided to use these native promoter elements to drive the Medicago Nod factor signaling genes in non-legume species.

**Table 1:** Nodulation of Medicago mutants complemented with the corresponding gene.

Gene complemented	Mutant	Nodule number
Control	wt	13.9 (+/−4.6)
*MtLYK3*	B56 (lyk3)	13.1 (+/−5.5)
*MtNFP*	C31 (npf)	6.2 (+/−3.8)
*MtDMI1*	B129 (dmi1)	10.9 (+/−5.7)
*MtDMI2*	TR25 (dmi2)	12.4 (+/−6.1)
*MtDMI3*	TRV25 (dmi3)	11.7 (+/−6.0)
*MtNSP1*	nsp1-1	14.2 (+/−8.4)
*MtNSP2*	nsp2-2	13.8 (+/−6.3)

The following mutants have been complemented. Nodules were counted 21 days after inoculation with *S. meliloti* strain 2011.

The full length *MtNIN* gene was cloned including a 3.5 kb 5′-upstream region. To detect its transcription, it needs to be induced and analyzed by qRT-PCR. To verify whether the cloned *MtNIN* gene included the necessary Nod factor responsive elements, a GUS-reporter construct with a similar 5′ upstream region was made. This MtNINp-GUS construct was introduced in Medicago roots and GUS activity was studied in response to Nod factors (3 h) and *S. meliloti* (24 h). Strong GUS activity upon both treatments could be detected in the susceptible zone of the root, demonstrating that the promoter region cloned contained the regulatory elements responsive to early Nod factor signaling.

### Gene Stacking in pHUGE-Red

**Figure 3:**
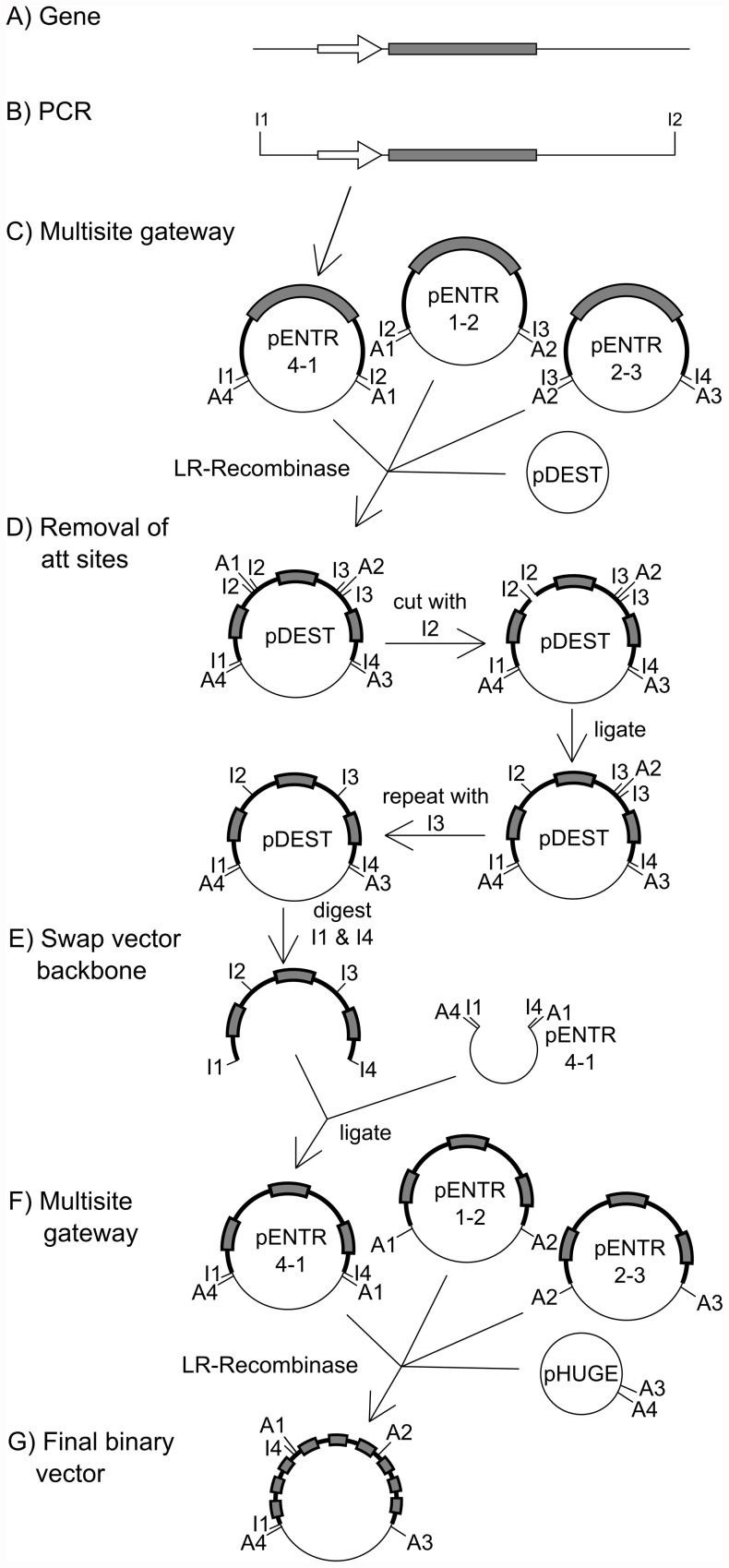
Construction of pHUGE-MtNFS and pHUGE-LjMtNFS. A: A Gene of interest, including 2–3 kb of up- and 2 kb downstream genomic region. B: The genomic region was cloned by PCR using primers introducing the endonuclease recognition sites I1 & I2. PCR products were cloned into pENTR vectors. Restriction sites I1 & I2, I2 & I3 or I3 & I4 introduced at the start and the end of different genomic regions have to be unique within the three vectors combined in step D. C: The resulting vector, containing the full length gene flanked by *att* recombination sites (A4 & A1) is combined with two alternative entry vectors and a pDEST vector. Both vectors are created in a similar fashion and contain an additional gene of interest flanked by the restriction sites I2 & I3 or I3 & I4. D: *att* recombination sites are removed in two rounds of digestion (I2, I3), heat inactivation of the restriction enzyme and subsequent re-ligation. E: The pDEST backbone is digested using the endonuclease recognition sites I1 & I4 and ligated into a predigested entry vector (either pENTR4-1, pENTR1-2 or pENTR2-3) without purification. Transformed library efficient DH10b cells are selected on kanamycin. Each resulting entry vector contains three DNA fragments. F: Finally, these vectors were combined into pHUGE-Red using multisite gateway. G: Final binary vector containing up to nine genes.

The MultiSite Gateway cloning cassette as introduced into pHUGE-Red allows cloning of three entry clones by recombination. So, in a single cloning step three genes of interest, each cloned into a compatible entry vector, can be recombined into pHUGE-Red in a single LR recombinase reaction. To enable cloning of up to nine genes we developed a strategy consisting of two rounds of LR recombination and a backbone swap (Fig. 3).

The genes of interest, seven Medicago- and two Lotus nodulation genes, were amplified by PCR. An unique endonuclease restriction site was introduced at the 5′ end of each PCR oligonucleotide designed (Fig. 3; step B). These endonuclease restriction sites were required to remove the *att* recombination sites during a later step of the cloning procedure. Amplified genes were cloned in one of the three available MultiSite Gateway compatible entry clones; pENTR4-1R, pENTR1-2, pENTR2R-3. The restriction site introduced by the reverse PCR primer of the gene cloned in pENTR4-1R was identical to the restriction site introduced by the forward PCR primer of the gene cloned into pENTR1-2 (Fig. 3; step C). Likewise, the restriction site introduced by the reverse PCR primer in the gene cloned in pENTR1-2 was identical to the restriction site present in the forward PCR primer of the gene cloned into pENTR2R-3 (Fig. 3; step C). Using MultiSite Gateway the three entry vectors were combined into a single destination vector. Subsequently *att* recombination sites, each flanked by a pair of unique restriction sites, (Fig. 3, step D) have been removed by two following rounds of restriction-ligation based cloning. The 30 kb DNA fragments were not purified at this step to avoid shearing. Ligation was efficient even at this size due to the absence of an insert. Each of these vectors can be used three times resulting in a maximum of nine genes. This created three destination vectors each containing three genes. The backbones of these destination vectors were exchanged for the backbones of one of the three entry clones (pENTR4-1R, pENTR1-2, pENTR2R-3) by restriction-ligation based backbone swapping. A second LR recombinase driven reaction was used to combine the three gene clusters into a single pHUGE-Red or pHUGE-RedSeed binary vector. Using this strategy two binary vectors were created; pHUGE-MtNFS (accession nr. JN874482), containing eight genes (and one empty ENTR clone) *MtLYK3*, *MtNFP*, *MtDMI1*, *MtDMI2*, *MtDMI3*, *MtNSP1*, *MtNSP2* and *MtNIN* and pHUGE-LjMtNFS (accession nr.: JN874483) for which *MtLYK3* and *MtNFP* are replaced by *LjNFR1* and *LjNFR5* (Table 2).

**Table 2:** Plasmids used for transformation of strawberry, poplar, tobacco and tomato.

	NOD factor signaling genes from RB to LB	Length of T-DNA	Accession Nr.
pHUGE-MtNFS	*MtNFP, MtLYK3, MtDMI2, MtDMI3, MtDMI1,* *MtNSP2, MtNSP1, MtNIN*	72 kb	JN874482
pHUGE-LjMtNFS	*LjNFR5, LjNFR1, MtDMI2, MtDMI3, MtDMI1,* *MtNSP2, MtNSP1, MtNIN*	74 kb	JN874483

### One-step *Agrobacterium*-mediated Transformation of Eight Genes into Non-legume Species

**Figure 4:**
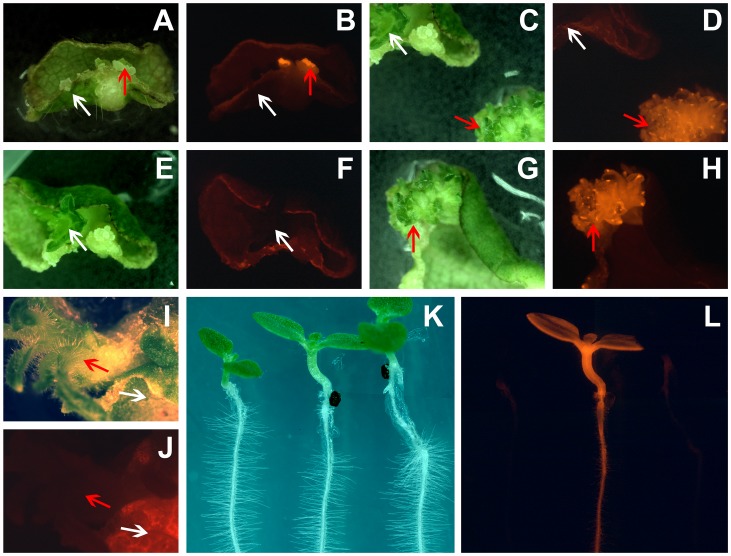
Selection of transformed lines. Tissues are analyzed by bright field microscopy (A, C, E, G, I, K) and fluorescent microscopy using DsRED1 filter settings (B, D, F, H, J, L). A+B: Discrimination between transformed strawberry calli (red arrow) and untransformed calli (white arrow) based on expression of DsRED1. C+D: Outgrowth of transgenic- (red arrow) and non-transgenic strawberry plants (white arrow). E+F: Non-transgenic strawberry plant (white arrow). G+H: Transgenic strawberry plant (red arrow). I+J: Outgrowth of marker free tobacco plantlet, (red arrow) on a DsRED1 expressing leave disk (white arrow). K+L: Mosaic picture showing wild type tobacco (left), transformed tobacco expressing DsRED1 before (middle) and after marker gene removal (right).

For the transfer of the Nod factor signaling genes we selected five non-legume species; namely Arabidopsis, tobacco (*Nicotiana tabacum*), tomato (*Solanum esculentum*), strawberry (*Fragaria×ananassa*) and poplar (*Populus tremula*×*P. alba* hybrid). Tobacco and tomato, both Solanaceae, are phylogenetically most distinct to legumes, whereas poplar and strawberry together with legumes are part of the Fabids clade. Arabidopsis, as a member of the Malvids clade is phylogenetically positioned between these two clades. For tobacco, tomato, strawberry and poplar efficient transformation regeneration protocols are available (see material and methods). Transformation of these species was initiated using the binary vectors pHUGE-MtNFS and pHUGE-LjMtNFS employing *Agrobacterium tumefaciens* strain AGL1, which lacks recombinase activity (recA^-^), thereby enhancing stability of large binary plasmids [44]. Expression of DsRED1 allowed early identification of transgenic calli that subsequently could be regenerated to transgenic plants (Fig. 4, A–H). Stable lines were obtained for all species. In case of Arabidopsis we employed the floral dip transformation method [21]. For both constructs transformation of Arabidopsis was attempted twice but no transgenic seeds could be found. However, several transgenic Arabidopsis plants could be created using a smaller construct containing only *MtNFP*, *MtLYK3* and *MtDMI2*, proving the functionality of pHUGE-RedSeed.

To test the integrity of the transferred T-DNA we employed a PCR strategy that detects the seven artificial border regions between two stacked genes (Table S2). These analyses showed that in case of poplar, strawberry, tobacco and tomato successful transfer of the complete T-DNA had occurred in more than 50% of the transgenic lines analyzed (Table 3). Lines containing only a partial T-DNA lack in most cases genes flanking the right T-DNA border probably due to selection on marker genes that are located close to the left T-DNA border (Table S2). The observation that half of the lines have a deletion in the transferred DNA in concert with the size of the transferred DNA makes it unlikely that in the complete lines more than one copy was integrated into the genome.

**Table 3:** Transformation efficiency of pHUGE-Red.

Construct	Plant species	Transformation efficiency	Recombination efficiency
pHUGE-LjMtNFS	Tobacco	53% (10/19)	70% (7/10)
	Poplar	67% (2/3)	100% (1/1)
	Strawberry	50% (9/18)	67% (6/9)
	Tomato	100% (2/2)	NA
pHUGE-MtNFS	Tobacco	65% (11/17)	91% (10/11)
	Poplar	33% (1/3)	100% (1/1)
	Strawberry	60% (9/15)	100% (9/9)
Average		54% (42/77)	83% (34/41)

Transformation efficiency is given for the lines containing eight legume Nod factor signaling genes and three selection markers. Recombination efficiency is given for lines with the marker genes removed by recombination in a second round of regeneration. Brackets indicate absolute numbers of marker removed plant lines compared to over all treated plant lines.

### Marker Gene Removal

To test R/Rs recombinase-based marker removal system we regenerated transgenic lines of poplar, tobacco and strawberry in the presence of dexamethasone. 5′-Fluorocytosine was used to select plants from which the cassette containing *codA-nptII*, *DsRED1* and the R recombinase was recombined out. Whether recombination indeed had occurred was confirmed by PCR. For each of the transgenic lines, three plants were tested for successful recombination. To this end, two PCR reactions were performed using: (I) a primer pair that results in an amplicon when removal of the box containing the marker genes has occurred and (II) a primer pair that results in an amplicon when the box is still present. Furthermore, the presence of the eight Nod factor signaling genes was re-confirmed. Actual recombination could be triggered in 80% of all lines for which it was initiated (n = 41, Table 3). Initially recombination is visualized by the loss of red fluorescence (Fig. 4, I–L). All the lines tested had recombined out the box containing the marker genes, but still contained the eight Nod factor signaling genes.

### Gene Expression of Nod Factor Signaling Genes in Non-legumes

**Figure 5:**
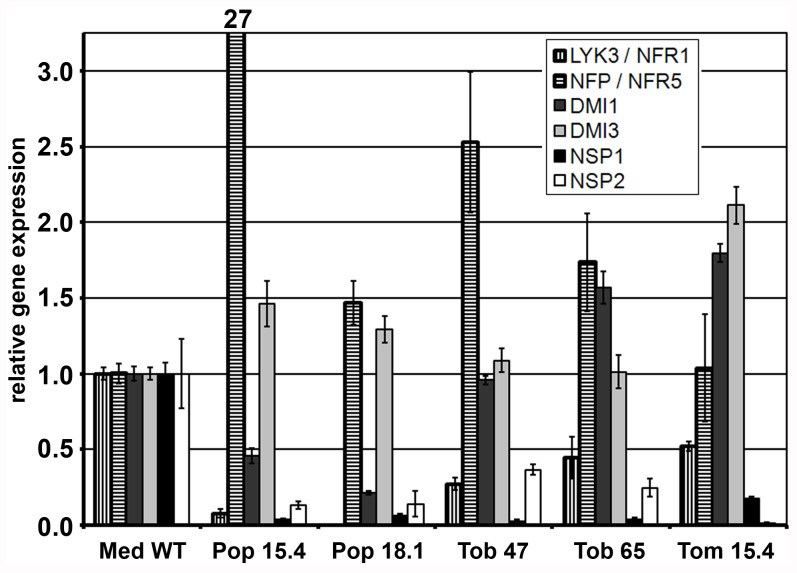
Relative gene expression of legume Nod factor signaling genes in trans. Expression has been studied in transgenic poplar (line Pop 15.4 and Pop 18.1), tobacco (line Tob 47 and Tob 65) and tomato (Tom 15.4). Gene expression was compared to native gene expression in Medicago (Med WT) which was set to 1. *MtDMI2* has been used as interspecies reference gene. Error bars show variation between three technical replicates.

To functionally characterize the generated lines, the expression of transgenes was studied first. Thereby we focused on poplar, tobacco and tomato as contaminants present in our strawberry RNA samples, likely sugars and/or phenolic compounds, prohibited further analysis. Expression of seven of the genes introduced, all except *MtNIN*, was verified using quantitative RT-PCR (qPCR). In all transgenic lines tested expression of all seven transgenes was detectable. Comparing relative expression of genes between species is difficult due to the lack of inter-species reference genes. To be able to do so, quantitative expression levels of the Nod factor signaling genes as estimated by qPCR were normalized based on *MtDMI2* expression. Of the genes introduced *MtDMI2* is highest expressed in all species, displaying similar Cq values. Normalization against *MtDMI2* indicated that the relative expression ratios differ between the lines (Fig. 5). Generally, the expression ratios for most genes showed to be comparable to Medicago, though, typical outliers are *MtNFP* in poplar (line 15.4) which showed to be relatively high expressed and *MtNSP2* in tomato (line 15.4) for which the expression is generally lower when compared to *MtDMI2* (Fig. 5). The same holds true for *MtNSP1* which is low expressed in all lines.

### Phenotypic Characterization

As a first test we determined whether any nodule-like structures could be formed on any of the transgenic lines, including strawberry, upon bacterial inoculation with Nod factor producing rhizobial strains. The microsymbiont of Medicago *-S. meliloti*- has a relatively small host range, and therefore intracellular hosting of this bacterium might ultimately not be possible in non-legumes. In contrast to *S. meliloti*, *Sinorhizobium* sp. NGR234 can nodulate over 110 legume genera (including Lotus) as well as the non-legume species of the Parasponia genus [45]–[46]. Adjustment of host range specificity by introduction of foreign Nod Factor receptors has been shown previously [39]. Therefore *S. meliloti* as well as *Sinorhizobium sp.*NGR234 were applied in this experiment. However, for none of the transgenic lines nodule-like structures could be observed 21 days post inoculation. Therefore we decided to analyze the transformed non-legume lines specifically for early Nod factor induced responses. We focus on molecular responses (*MtNIN* induction) and morphological responses.

**Figure 6:**
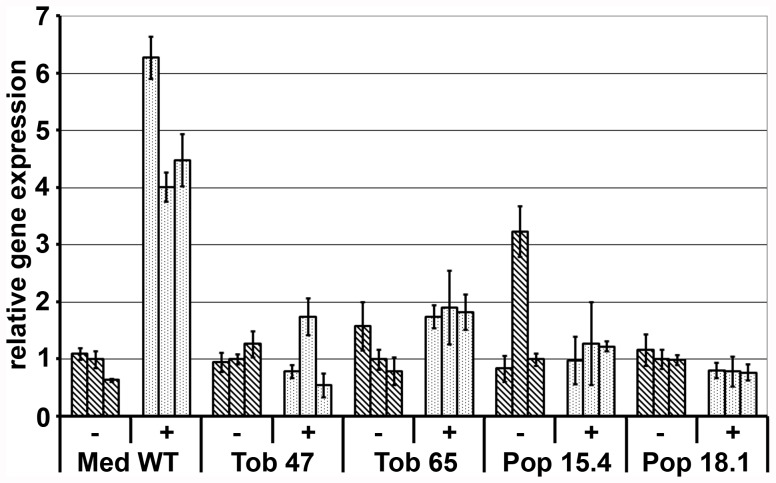
*MtNIN* induction upon Rhizobium application in legumes and non-legumes. Induction of *MtNIN* 24 h after exposure to flavonoid stimulated, compatible rhizobium bacteria (+) compared to water control (−) in Medicago (Med WT), transgenic poplar (line Pop 15.4 and Pop 18.1) and transgenic tobacco (line Tob 47 and Tob 65). Poplar *PtACT2* & *PtUBQ* and tobacco *NtGAPDH* were used as reference genes. Three biological replicates are shown for each line. Error bars show variation between three technical replicates.

Early Nod factor signaling resulting in division of cortical cells involves induction of the expression of the putative transcriptional regulator MtNIN. The functionality of the Nod factor signaling cascade after introduction of the eight legume genes can be tested by quantification of the level of *MtNIN* expression in response to bacterial Nod factors. In Medicago induction of *MtNIN* can be detected 3 hours following application of *S. meliloti* Nod factor, whereas 24 hours post inoculation with Nod factor producing rhizobium bacteria an average up regulation of 6 to 8 fold can be detected. *MtNIN* induction 24 hours after application of *S. meliloti* Nod factor producing rhizobia has been analyzed in transgenic lines of poplar, tobacco and tomato. Two lines per construct and species have been tested - when available. Induction is compared with *MtNIN* induction of Medicago wild type. In contrast to Medicago no induction of *MtNIN* could be detected in any of the lines tested (Fig. 6). This shows *MtNIN* induction cannot be triggered upon bacterial inoculation in any of the transgenic lines.

These analyses suggest that the Nod factor signaling cascade is interrupted at an earlier stage of signal transduction. To narrow down the point where Nod factor induced signal transduction is blocked, we studied symbiosis specific responses upstream of *MtNIN* induction; root hair deformation. Root hair deformation is dependent on Nod factor receptors and further facilitated by the *MtDMI* genes [35] [47]. We have tested *S. meliloti* Nod factor induced root hair deformation for two species; tobacco line 47 and line 65 as well as tomato line 15.4. No deformations could be detected for these lines (3–12 h post Nod factor application).

## Discussion

Here we present an easy to use one-step plant transformation system to transfer multiple genes at once. Two novel binary vectors, named pHUGE-Red and pHUGE-RedSeed, have been created which facilitate modular cloning of large DNA fragments. Both vectors contain two selectable markers; *nptII* and a non-destructive fluorescent marker *DsRED1* to verify T-DNA transfer. Depending on the vector *DsRED1* is placed either under control of a strong constitutive promoter (pHUGE-Red) or a seed coat specific promoter (pHUGE-RedSeed). For efficient removal of the marker genes after successful transformation, they are flanked by Rs recombination sites. We used these vectors successfully in four different species to transfer eight symbiotic legume genes.

The transfer of eight genes in a single transformation step requires a binary vector capable of straightforward cloning of relatively large DNA fragments and efficient selection of transgenic material. A MultiSite Gateway cassette was introduced into the vector to simplify cloning. This allows one-step cloning of three genes. By backbone swapping and removal of *att* recombination sites we reverted the destination clones back into entry clones, enabling a second round of multisite gateway cloning and making stacking of nine genes possible. Alternatively, pHUGE-Red vectors can be used in Multiround Gateway projects if a step by step integration of genes is preferred to the parallel approach of this study [14]. The vector also allows several rounds of plant transformation, as the makers genes can be removed efficiently. Marker genes have been embedded in an Rs recombination cassette. We showed the effectiveness of the recombination system for three plant species, poplar, strawberry and tobacco. In 80% of all re-regenerated lines the selectable markers had been removed, whereas the remaining of the inserted T-DNA was not affected.

Recombination based cloning is size independent, therefore, in theory fragments of any length can be recombined in. The effectiveness of this method was demonstrated by combining three fragments, each of a size between 20–35 kb, into a binary vector resulting in a 74 kb T-DNA (including selection markers). Transfer and selection efficiency was monitored by the use of *DsRED1* driven by the constitutive Arabidopsis *AtUBQ10* promoter. Four different plant species were successfully transformed by regeneration of transgenic calli using this vector. The presence of DsRED1 in transgenic tissue guided the selection of transgenic calli and/or plantlets at any stage of the regeneration procedure and eased the selection process. Due to the low level of background fluorescence in the DsRED1 emission spectrum the number of false positives could be kept to a minimum [23]. Attempts were made to transform Arabidopsis employing pHUGE-RedSeed variants of the plasmids. However, no transgenic plants were obtained by using plasmids containing a T-DNA of over 70 kb. A major difference was the floral dip method employed for transformation [21] which was used to transfer T-DNA of 84 kb [9], but possibly needs optimization in our hands. Transfer of a smaller insert (35 kb) was successful using this method (data not shown), illustrating that pHUGE-RedSeed is functional.

Genomic DNA-gel blots are the gold standard to prove copy number and integrity of the transferred DNA. However, in the case of large constructs this approach comes to its limitations. A genomic DNA-gel blot with a probe for the entire T-DNA would result in a very high number of overlapping bands of a large size range making it difficult to identify them correctly. If the probe only detects a specific, smaller region, a high number of blots and probes would be needed to evaluate the entire transgene, making screening difficult. Therefore we decided to amplify the artificial border regions between two stacked genes by PCR. This method allowed fast screening of many samples and provided a good overview of the state of the integrated DNA. For each species transformed we also identified lines that contained only a partial T-DNA and due to the location of the selectable markers close to left T-DNA border sequence, mainly regions close to the right T-DNA border was truncated. To limit recombination events in Agrobacterium and thereby enhancing plasmid stability we used a strain lacking recombinase activity (recA-), as suggested by Shibata and Liu [44]. All together this led to 50% efficiency of full length T-DNA integration in DsRED1/nptII positive lines. This is a remarkable result, taken into account that wild type T-DNA is 10–30 kb in size, while the T-DNA in this experiments is 74 kb in size. Due to the large size and the high amount of incomplete transferred transgenes, it is unlikely that more than one copy is integrated. Of the plants generated, tobacco and tomato were propagated generatively. In no case transgene instability has been observed. Possibly, the relative stability of the construct used is underpinned by cloning each transgene under its native promoter and terminator recombination thereby limiting the presence of homologous sequences.

Eight Medicago genes essential for nodulation were cloned in their native genomic context, including native promoter and terminator regions. The constructs showed to be sufficient for complementation of the corresponding mutant, suggesting that all cis regulatory elements (CREs) essential to coordinate transcriptional activity as well as Nod factor signaling in legumes are present. The functionality of these CREs in non-legumes has been studied in two ways. First we conducted promoter::GUS studies in tomato and Arabidopsis. Secondly, we have validated expression of the introduced genes in the different transgenic species using quantitative RT-PCR. The promoter-reporter experiments revealed for all genes a global spatial expression pattern in tomato and Arabidopsis similar to Medicago. Nonetheless, qPCR analysis of the trans genes in poplar, tomato and tobacco indicated variation in expression levels compared to Medicago. Generally expression of the trans genes was detectable, though in all species the expression of *MtNSP1* and *MtNSP2* showed to be relatively low, whereas a relatively high expression of *LjNFR5*/*MtNFP* was found in poplar. We realize that comparison of relative gene expression is difficult between species due to the lack of inter-species reference genes. By using *MtDMI2*, the highest expressed gene in all species, as an internal reference, we have made an attempt to address this issue. Overall expression of these genes suggests that regulatory elements required for proper expression are conserved in legumes and non-legumes and transcription factors required for binding these elements are present in non-legumes.

The four transgenic species expressing legume Nod factor signaling genes were studied for Nod factor triggered morphological responses (root hair deformations and cortical cell divisions) and molecular responses (induction of *MtNIN* expression). In no case symbiotic responses could be observed. Therefore we conclude that transfer of seven legume Nod factor signaling genes and *MtNIN* is insufficient to trigger Nod Factor induced responses in non-legumes. A number of reasons can explain the lack of response. The differences in quantitative expression could be the reason for a non-functioning signaling cascade. Especially, the *LjNFR1*/*MtLYK3* gene is relatively low expressed when compared to Lotus or Medicago. On the other hand, a weak allele in Medicago of this gene (*hcl-4*), which effectively has less than 10% of the *MtLYK3* transcript displays a significant weaker phenotype when compared to complete knock outs, and occasionally makes functional nodules [48]. This suggests that a significant lower expression level of this gene does not terminate Nod factor signaling. Besides misexpression of the introduced genes, two alternative explanations can be given for the lack of symbiotic responses in the non-legume species. First, the selected set of genes that have been transferred is incomplete. The genes were selected at the start of this project based on results of mutant screenings. All these genes are essential for Nod factor induced nodule primordium formation and gene expression. Currently, several additional genes have been identified in Medicago and/or Lotus. The knockout phenotypes in Nod factor signaling of these genes however are less severe. Second, more generic hormone signaling pathways that are essential for root nodule formation could be differently regulated in legumes than in non-legumes. For example, physiological studies as well as genetic analyses have revealed the importance of cytokinin for root nodule formation [49]–[57]. Although, phylogenetic studies of the genes encoding key elements of the cytokinin phosphorelay signaling pathway did not reveal any legume specific orthology group [58], differential expression of these genes could be the cause of the difference in response. Due to the option of marker removal the set of transgenic lines generated within this project can be used as a stepping stone in the process of creating non-legumes containing a larger set of genes essential for nodulation.

Although to the expression of these seven key regulators is insufficient to trigger any Nod factor induced symbiotic responses including the transcriptional activation of *MtNIN*, the vectors pHUGE-Red and pHUGE-RedSeed proved to be valuable tools for the strait forward generation of transgenic plants containing several *trans* genes. BIBAC and pYLTAC7 are two alternative binary vectors specifically designed for the transfer of large DNA fragments. Although both vectors are publicly available for more than a decade now, only a limited number of successful experiments have been reported making use of these vectors. We cannot judge whether the hesitation of using this technology is due to difficulties related to cloning, the transformation process itself or stability of the T-DNA over multiple rounds of propagation. Furthermore, it is suggested that transfer of large DNA fragments into plants by the use of *A. tumefaciens* is problematic [59]. Using the pHUGE-Red vector and the published transformation protocols, no major difficulties were encountered during the course of the experiments. This fact encouraged us to think that the transfer of large DNA fragments is rather straightforward. Based on our good experiences we would like to share these vectors with the scientific community and encourage their use. Furthermore we hope to discuss the problems which give the transfer of large DNA fragments a bad reputation.

## Supporting Information

Table S1 Primer Sequences. Primers used for expression analysis by qPCR. All primers were designed using primer3plus [22].
(DOC)

Table S2 Identification of transgenic lines. Analysis of the different transgenic lines by PCR to verify the integration of all eight genes transferred.
(DOC)

Data S1 Cloning of pHUGE. Detailed description of the cloning strategy of the pHUGE-Red binary vector.
(DOC)

Data S2 Cloning of pHUGE – Sequences. Fasta file containing all sequences used for cloning pHUGE-Red.
(FAS)
