# Supplementary material for: One-Step Agrobacterium Mediated Transformation of Eight Genes Essential for Rhizobium Symbiotic Signaling Using the Novel Binary Vector System pHUGE
Source: PLoS One. 2012 Oct 24;7(10):e47885. doi: 10.1371/journal.pone.0047885 (PMC3480454; doi:10.1371/journal.pone.0047885)
Supplement: Table S1 — Primer Sequences. Primers used for expression analysis by qPCR. All primers were designed using primer3plus [22]. (DOC) [file pone.0047885.s001.doc]

**Table S1:**

| **Gene** | **Forward primer** | **Reverse primer** |
| --- | --- | --- |
| *MtNFP* | GCTCTAAGTTTGGCTTCTTTGGCAGTA | TGGTTCAGATGATGGTTGGTTGA |
| *MtLYK3* | TGATTCTGTTCTCAAGATGGCTCAA | CAATCTTCAGTTGGTGATGAAAGTGTC |
| *MtDMI1* | TTATTTGCGGAGGAGGGGAAC | CAATCTCCTTTCTTGTACGACCCCTA |
| *MtDMI2* | GGAGCTTGGTTGAATGGGCTAA | TGCTACTTCCACAACTCTCCACAATG |
| *MtDMI3* | TTGTTTGACAACAACCGTGATGG | CATACATCTGGAAGCACAAACGAAGA |
| *MtNSP1* | GCGATTTCGCCACTGGATTC | CAGCCTCGCCTTCCATCATT |
| *MtNSP2* | GGCCTAGAATTGCAGGCTCGT | TTGCAAAGCTCACCGGAACTC |
| *MtNIN* | GGGAGAAAGTCCGGGGACAA | GACACACACCGATGCTCTTTGC |
| *MtGAPDH* | CCCTTCATCTTGTCCTTCGTCTG | CACCTCCAATGTAATGGTCTTTCC |
| *LjNFR5* | CCCCATGATGCCAAAAATCG | TCTCCCACATATCCTTCCACAGC |
| *LjNFR1* | GAATCTGGTGCGCTTGATTGG | CCATGGCAATGGTTCTTTACCTG |
| *PtACT2* | CCCATTGAGCACGGTATTGT | TACGACCACTGGCATACAGG |
| *PtUBQ* | GTTGATTTTTGCTGGGAAGC | GATCTTGGCCTTCACGTTGT |
| *PtNIN1* | AAGAGAACACACAGTGATGCCGC | GTCTCTGGATTTGGAAGGTCGCC |
| *PtNIN2* | CGGACCAAGACAGCGATCCAG | GTAGCATCTTTGAGGCTCCCTGC |
| *PtNIN3* | TAGGAAGCATTTGCCCGACCAC | GCTCCCTTGGCACCTTGGAC |
| *PtNIN4* | TTGGCAGACATCTCTCATTTGGC | CAAACAGCGTCTTTGAGGCTCC |
| *AtACT2* | CTCTCCTTGTACGCCAGTGGTC | TAAGGTCACGTCCAGCAAGGTC |
| *AtNIN1* | CAACCCAAACTGTCCATGTGC | AAGCCACCAAGTTGTTGCTCTTC |
| *AtNIN2* | TGTCTGCAGAACTACACCGAGTC | ACGTTCAGGGAAATGATGAGAAG |
| *AtNIN3* | TCAGCACAGGGAACTGCTG | TTCAGCCATCAGAGTGGTTACAG |
| *NtGAPDH* | GATGCTCCGATGTTTGTTGTCG | GGGAGCAAGGCAATTTGTGG |

**Supplemental Table S1:** Primers used for qPCR.
