# Supplementary material for: One-Step Agrobacterium Mediated Transformation of Eight Genes Essential for Rhizobium Symbiotic Signaling Using the Novel Binary Vector System pHUGE
Source: PLoS One. 2012 Oct 24;7(10):e47885. doi: 10.1371/journal.pone.0047885 (PMC3480454; doi:10.1371/journal.pone.0047885)
Supplement: Table S2 — Identification of transgenic lines. Analysis of the different transgenic lines by PCR to verify the integration of all eight genes transferred. (DOC) [file pone.0047885.s002.doc]

**Supplemental Table S2:**

**A: Strawberry**

**B: Tobacco**

**C: Poplar**

**D: Tomato**

**Supplemental Table S2:** Identification of transgenic lines. Border regions between two genes are amplified by PCR. Positive PCR is indicated by X.
