# Supplementary material for: One-Step Agrobacterium Mediated Transformation of Eight Genes Essential for Rhizobium Symbiotic Signaling Using the Novel Binary Vector System pHUGE
Source: PLoS One. 2012 Oct 24;7(10):e47885. doi: 10.1371/journal.pone.0047885 (PMC3480454; doi:10.1371/journal.pone.0047885)
Supplement: Data S1 — Cloning of pHUGE. Detailed description of the cloning strategy of the pHUGE-Red binary vector. (DOC) [file pone.0047885.s003.doc]

Description of the cloning of pHUGE-Red and pHUGE-RedSeed. Matching sequences are in fasta format in file Cloning_of_pHuge_seq.fas

**1. Introduction of spectinomycin resistance in pYLTAC7**

**A:** A part of **pYLTAC7** was PCR amplified using primers

caccattccggacccgggctcgagtcgacTACGATCCGCTTCGGCAGG

CGCGTTATCACTGGATAGCATCG

and cloned into pENTR D/Topo resulting in pINTER1. The forward primer introduced the restriction sites Bsp EI, Sma I, Xho I and Sal I.

**B:** The spectinomycin resistance gene of **pKGW** was PCR amplified using primers

CACCGTCGACCCGGGatcagtccgggacggcgtcagcgggagagccgttg

ATGTCGACCtggtcatgcatgatatatctcccaatttgtgtagg

and cloned into pENTR D/Topo resulting in pINTER2. The forward primer introduced the restriction sites Sal I and Sma I, the reverse primer introduced Sal I.

**C:** The Sal I fragment of pINTER2 containing the spectinomycin resistance was cloned introduced at the SalI site in vector pINTER1, resulting in vetor pINTER3.

**D:** The PacI - Apa I fragment of **pYLTAC7** was isolated and cloned into a modified pENTR clone creating clone pINTER4. In this fragment the Aat II site is unique.

**E:** The Apa I - Bsp EI of pINTER3 was cloned into pINTER4 fragment of which was digested Apa I - Ngo MIV creating plasmid pINTER5.

**F:** The Pac I - Apa I fragment of pINTER5 was used to replace the respective region in **pYLTAC7** (using a partially digest of Pac I), creating pINTR6.

**2. Modification of the transfer cassette in pYLTAC7**

**A:** A dimer was created using the primers:

taaggccggccgcggccgcaagcttggcgcgccATAA

ggcgcgccaagcttgcggccgcggccggccttaAT

The vector pINTR6 was digested Pac I (partially) and I-Sce I. The dimer was cloned in forming clone pINTR7. The dimer introduces the restriction sites Fse I, Sac II, Not I, Hind III and Asc I. The restriction sites Fse I, Not I, Hind III and Asc I are unique in pINTR7.

**B:** A part of **pKm43GW** was PCR amplified using primers:

CACCAGATCTGACGTCacgacggccagtgaattatc

GATATGGCGCGCCCACCCTTTAAAGCttcacacaggaaacagctatga

and cloned into pENTR D/Topo resulting in pINTER8. The forward primer introduced the restriction sites Bgl II and Aat II, the reverse primer site Asc I.

**C:** A part of **pMF1** was PCR amplified using primers:

CACCTAAGGCCGGCctacgatttgatgaaagaatgaatta

TCGTGACGTCAGATCTtatcactgtggacgttgatgaaaga

and cloned into pENTR D/Topo resulting in pINTER9. The forward primer introduced the restriction sites Fse I, the reverse primer Spe I, Sac I, Xho I, Kpn I and Eco RI.

**D:** The Eco RI - Asc I fragment of **pMF1** was cloned into pINTR 9 which was digested Eco RI - Asc I as well forming pINTR10.

**E:** The Bgl II - Asc I fragment of pINTR8 was cloned into pINTR 10 which was digested Bgl II - Asc I as well forming pINTR11.

**F:** A part of **pKGW-RR** was PCR amplified using primers:

CACCggtacccggggatccct

GTTACTAGTgacgtcgttgtggttggtgct

and cloned into pENTR D/Topo resulting in pINTER12. The reverse primer introduced a restriction site Spe I.

**G:** The Kpn I - Spe I fragment of pINTR12 was cloned into pINTR 11 which was digested Kpn I - Spe I as well forming pINTR13.

**H:** The Fse I - Asc I fragment of pINTR13 was cloned into pINTR 7 which was digested Fse I - Asc I as well forming the final clone **pHUGE-Red**.

**I:** A part of **pFLUAR101** was PCR amplified using primers:

CACCggtaccgagctcgaattcg

ATAGACGTCGGGCCCATCGATGGCGCgctgattgcccttcaccgc

and cloned into pENTR D/Topo resulting in pINTER14. The reverse primer introduced a restriction site Aat II.

**J:** The KpnI - Aat II fragment of pINTR14 was cloned into **pKGW** which was digested KpnI - Aat II as well forming the final clone **pKGW - RedSeed**.

**K:** The KpnI - Aat II fragment of pINTR14 was cloned into **pBGW** which was digested KpnI - Aat II as well forming the final clone **pBGW - RedSeed**.

**F:** A part of pKGW- RedSeed was PCR amplified using primers:

CACCggtaccgagctcgaattcg

ACACTAGTgacgtcgggcccatcgat

and cloned into pENTR D/Topo resulting in pINTER15. The reverse primer introduced a restriction site Spe I.

**G:** The Kpn I - Spe I fragment of pINTR15 was cloned into pINTR 11 which was digested Kpn I - Spe I as well forming pINTR16.

**H:** The Fse I - Asc I fragment of pINTR16 was cloned into pINTR 7 which was digested Fse I - Asc I as well forming the final clone **pHUGE-RedSeed**.
